# Patient Preferences for Treatment of Psoriasis with Biologicals: A Discrete Choice Experiment

**DOI:** 10.1371/journal.pone.0129120

**Published:** 2015-06-09

**Authors:** Christian Kromer, Marthe-Lisa Schaarschmidt, Astrid Schmieder, Raphael Herr, Sergij Goerdt, Wiebke K. Peitsch

**Affiliations:** 1 Department of Dermatology, University Medical Center Mannheim, Heidelberg University, Mannheim, Germany; 2 Mannheim Institute of Public Health, Social and Preventive Medicine, Medical Faculty Mannheim, Heidelberg University, Mannheim, Germany; Kermanshah University of Medical Sciences, Islamic Republic of Iran

## Abstract

Treatment dissatisfaction and non-adherence are common among patients with psoriasis, partly due to discordance between individual preferences and recommended treatments. However, patients are more satisfied with biologicals than with other treatments. The aim of our study was to assess patient preferences for treatment of psoriasis with biologicals by using computer-based conjoint analysis. Biologicals approved for psoriasis in Germany were decomposed into outcome (probability of 50% and 90% improvement, time until response, sustainability of success, probability of mild and severe adverse events (AE), probability of American College of Rheumatology (ACR) 20 response) and process attributes (treatment location, frequency, duration and delivery method). Impact of sociodemographic and socioeconomic characteristics and disease severity on Relative Importance Scores (RIS) of each attribute was assessed with analyses of variance, post hoc tests, and multivariate regression. Averaged across the cohort of 200 participants with moderate-to-severe psoriasis, preferences were highest for avoiding severe AE (RIS = 17.3), followed by 90% improvement (RIS = 14.0) and avoiding mild AE (RIS = 10.5). Process attributes reached intermediate RIS (8.2–8.8). Men were more concerned about efficacy than women (50% improvement: RIS = 6.9 vs. 9.5, p = 0.008; β = -0.191, p = 0.011 in multivariate models; 90% improvement: RIS = 12.1 vs. 15.4, p = 0.002; β = -0.197, p = 0.009). Older participants judged the probability of 50% and 90% improvement less relevant than younger ones (50% improvement: Pearson’s Correlation (PC) = -0.161, p = 0.022; β = -0.219, p = 0.017; 90% improvement: PC = -0.155, p = 0.028; β = -0.264, p = 0.004) but worried more about severe AE (PC = 0.175, p = 0.013; β = 0.166, p = 0.082). In summary, participants with moderate-to-severe psoriasis were most interested in safety of biologicals, followed by efficacy, but preferences varied with sociodemographic characteristics and working status. Based on this knowledge, physicians should identify preferences of each individual patient during shared decision-making in order to optimize treatment satisfaction, adherence and outcome.

## Introduction

Psoriasis is one of the most common chronic-inflammatory diseases of the skin and joints with high impact on emotional and social well-being, life course and occupational career [1]. The well-being of affected patients is not only influenced by the psoriasis itself, but also by its management [2,3]. To identify an effective treatment with reasonable risks and costs, physicians often chose a stepwise approach starting with topical and phototherapy, escalating to traditional systemic medication and ending with biologicals [4].

Biologicals approved as second-line treatments for refractory psoriasis in Germany consist of the TNF antagonists etanercept, adalimumab and infliximab and the interleukin 12/23 antagonist ustekinumab. All of them have a favourable benefit-risk profile [4,5] but they possess some differences in response rates, rapidity of action and sustainability [6–8]. Chances of achieving reduction of the Psoriasis Area and Severity Index (PASI) by 50, 75 or 90% (PASI 50, 75 or 90 response rates) are higher for infliximab, ustekinumab and adalimumab than for etanercept [6]. Onset of action is fastest for infliximab, followed by ustekinumab and adalimumab [8]. Both TNF antagonists and ustekinumab are approved for psoriatic arthritis, but American College of Rheumatology (ACR) 20 response rates may be somewhat higher for TNF antagonists [9,10]. Moreover, the treatment process, i.e., the mode and frequency of application of each biological is different. Etanercept, adalimumab and ustekinumab are administered subcutaneously (etanercept and adalimumab with a pen or a prefilled syringe, ustekinumab with a prefilled syringe). Infliximab is given intravenously as infusion. Etanercept has to be applied once to twice weekly, adalimumab every two weeks, infliximab every eight and ustekinumab every 12 weeks.

Patients with psoriasis receiving biologicals are on average very satisfied with their treatment, whereas patients with other treatments report higher dissatisfaction, often caused by discordance between treatment requirements and individual needs [2]. This contributes to high rates of non-adherence [2,11,12]. One way to improve treatment satisfaction, adherence and thereby outcome is integration of patients’ preferences into shared decision-making. Patients’ preferences for psoriasis treatments were assessed in a number of studies and with different techniques, some of which originally stem from marketing research [13–17]. We previously performed conjoint analysis (CA), a method imitating the trade-off process typical of clinical decision-making, to elicit patients’ preferences for psoriasis treatments [18–21]. We showed that when faced with all kinds of treatment options including topical therapy, phototherapy, traditional systemic therapy and biologicals patients prioritize an efficient and convenient outpatient therapy [18]. The aim of the present study was to identify patients’ preferences for outcome and process attributes of biologicals and to study the impact of sociodemographic and socioeconomic factors and disease severity on these preferences.

## Materials and Methods

### Study participants

Preferences of patients with moderate-to-severe psoriasis for the attributes of biologicals were assessed in an open cross-sectional study at the Department of Dermatology of the University Medical Center Mannheim, Germany. All patients presenting to our outpatient department with moderate-to-severe psoriasis were pre-screened to determine their eligibility to participate in the study. Every eligible patient was asked to participate. Participants who had provided written informed consent were carefully examined and checked for inclusion and exclusion criteria. Inclusion criteria were age ≥18 years and moderate-to-severe psoriasis according to the criteria of the Committee for Medicinal Products for Human Use (CMPH), i.e., PASI≥10 in the course of the disease, involvement of head, palms or plantar surfaces, or psoriatic arthritis according to Classification of Psoriatic Arthritis (CASPAR) criteria with any skin involvement [22]. Diagnosis of psoriasis was based on clinical examination, combined with histology if the clinical diagnosis was questionable. Both patients with and without current antipsoriatic treatment were eligible for participation. Exclusion criteria were other diagnoses than moderate-to-severe psoriasis and inability to complete the survey due to difficulties with the German language or incapacity to understand CA exercises (i.e., failing to answer exercises with unambiguous scenarios presented for control). The study was performed according to the principles of the Declaration of Helsinki and approved by the Ethics Committee of the Medical Faculty Mannheim (Ethics Approval 2009-329E-MA, 22 October 2009; Amendment 27 September 2012).

### Data collection

Participants were assigned an identification code to access a computerized survey before clinical consultation. The first part contained information on sociodemographic characteristics (age [in years], gender and partnership [living with or without a partner]) and socioeconomic characteristics (working status [not working (including homemakers and retirees), working part-time, or working full-time] and net monthly household income [<1500 €, 1500-<3000 €, or ≥3000 €]) as well as the Dermatology Life Quality Index (DLQI).

In the second part, participants’ preferences for treatment of psoriasis with biologicals were explored using CA, generated and evaluated basically as described [18]. Seven key outcome attributes (probability of 50% improvement, probability of 90% improvement, time until response, sustainability of success, probability of mild adverse events (AE), probability of severe AE, and probability of ACR 20 response) and four process attributes (treatment location, frequency, duration, and delivery method) were selected (Table 1). Literature research including randomized controlled trials, guidelines, reviews and meta-analyses was performed to identify four realistic levels for each attribute that reflected characteristics of TNF antagonists and ustekinumab as closely as possible (e.g., [4–10; 23–26]). Attributes were assigned to two groups, each with six attributes, to prevent information overload. Treatment duration was included into both groups to enable same-scaled comparison across all attributes. The two attributes describing efficacy (probability of 50% and 90% improvement) were presented in different groups. The probability of 90% improvement was depicted as chance of almost complete clearance. Examples of discrete choice scenarios with attributes of group 1 and group 2 are provided in Table 2. The CA exercises did not contain a cost attribute, because treatment costs for biologicals are usually covered by health insurance in Germany.

**Table 1:** Outcome and process attributes and attribute levels used in the conjoint scenarios.

Outcome attribute	Level
Probability of 50% improvement	90–95%
	85–90%
	80–85%
	70–80%
Probability of 90% improvement	50–60%
	40–50%
	30–40%
	20–30%
Time until response	2 weeks
	4 weeks
	8 weeks
	12 weeks
Sustainability of therapeutic success^1^	20%
	15%
	10%
	5%
Probability of mild AE	50–70%
	30–50%
	10–30%
	<10%
Probability of severe AE	5–10%
	2–5%
	1–2%
	<1%
Probability of ACR 20 response	50–60%
	40–50%
	30–40%
	20–30%
**Process attribute**	**Level**
Treatment location	at home
	at a general practitioner’s office
	at a dermatologist’s office
	as an outpatient in a hospital
Treatment frequency	once to twice per week
	every two weeks
	every 4–8 weeks
	every 12 weeks
Delivery method	syringes into the subcutaneous fatty tissue administered by the patient
	syringes into the subcutaneous fatty tissue administered by medically trained persons
	injections into the subcutaneous fatty tissue with a pen administered by the patient
	infusions administered by a doctor
Treatment duration^2^	5 minutes
	15–30 minutes
	1 hour
	2–3 hours

^1^ Probability of loss of response within one year

^2^ per treatment session

AE: adverse events.

**Table 2:** Examples of discrete choice scenarios.

**Group 1**	
**Out of the two therapeutic options, please choose the one that you prefer.**	
***Option 1***	***Option 2***
The probability of reduction of my psoriasis by half is 90–95%.	The probability of reduction of my psoriasis by half is 80–85%.
The time until response is ca. 8 weeks.	The time until response is ca. 2 weeks.
The risk of mild adverse events (e.g., mild infections, injection-site reactions, headache, gastrointestinal symptoms, mild temporary change of laboratory parameters) is 35–50%.	The risk of mild adverse events (e.g., mild infections, injection-site reactions, headache, gastrointestinal symptoms, mild temporary change of laboratory parameters) is less than 10%.
The probability of improvement of psoriatic arthritis is 40–50%.	The probability of improvement of psoriatic arthritis is 20–30%.
My treatment will be administered every 2 weeks.	My treatment will be administered every 12 weeks.
Each treatment will take 1 hour.	Each treatment will take 5 minutes.
**Group 2**	
**Out of the two therapeutic options, please choose the one that you prefer.**	
***Option 1***	***Option 2***
The probability of almost complete clearing of my psoriasis is 20–30%.	The probability of almost complete clearing of my psoriasis is 50–60%.
The probability that the efficacy of the treatment will decrease within one year is ca. 5%.	The probability that the efficacy of the treatment will decrease within one year is ca. 10%.
The risk of severe adverse events (e.g., tuberculosis, other severe infections, severe intolerance reactions, autoimmune diseases) is less than 1%.	The risk of severe adverse events (e.g., tuberculosis, other severe infections, severe intolerance reactions, autoimmune diseases) is 2–5%.
My treatments will take place at home.	My treatments will take place as an outpatient in a hospital.
My treatments include injections into the subcutaneous fatty tissue with an easy-to-handle injection pen which I administer myself.	My treatments include infusions administered by a doctor.
Each treatment will take 5 minutes.	Each treatment will take 2–3 hours.

Participants were confronted with hypothetical treatment scenarios, designed by utilizing CBC/HB feature of commercially available CA software (http://www.sawtoothsoftware.com). This software created all possible combinations of attribute levels per group and randomly selected twelve pairs of scenarios of each group for each respondent by using a random-orthogonal method. In each experiment, respondents had to choose their preferred option with higher net utility. Three fixed experiments with one option being superior in every attribute were presented to each participant for control.

PASI was recorded by two investigators (C.K. and M.-L.S.). Part-worth utilities for each attribute level were computed using logit regression, with positive values indicating high utility and negative values representing disutility. The range between the highest and the lowest part-worth utility within one attribute was used to calculate the attribute’s Relative Importance Score (RIS) as fraction of one attribute’s range across the sum of all ranges. To compare results between attributes of group 1 and 2, attribute importance was translated into one list by matching the relative importance of treatment duration. Logarithmic transformations (Log_10_; for PASI, DLQI, probability of 50% improvement, time until response, probability of ACR 20 response, treatment frequency, duration, and delivery method) and square root transformations (for probability of 90% improvement, sustainability of success, probability of mild and severe AE, and treatment location) were applied to obtain normal distribution of the variables.

SPSS software was used for subgroup analyses. Associations of RIS with categorical variables were analysed by ANOVA (analysis of variance). Post hoc tests with Bonferroni correction were performed in cases of more than two subgroups. Pearson’s Correlations (PC) were used for continuous factors. For sensitivity reasons, analyses were repeated using untransformed variables with non-parametric tests (Kruskal-Wallis Test for ANOVAs and Spearman's rho for correlations). Significance was assumed at p≤0.05.

### Regression analysis

Multivariate linear regression analysis was performed to estimate independent associations between participants’ characteristics and RIS. Eleven models were created including age, gender, partnership, working status, DLQI, and PASI as independent variables and each attribute’s RIS as dependent variable. As monthly income did not significantly impact RIS according to descriptive analyses, this variable was not taken into account for the models. Standardized regression coeffients (β) were assigned to each independent variable, indicating the amount of change in RIS when varying one of the variables while holding the others constant.

## Results

239 patients were asked to participate; 210 met study criteria and provided written informed consent. Out of these, 10 had to be excluded, 9 because of difficulties with the German language and one due to inability understand the CA exercises. 200 participants completed the survey (57.5% male, mean age: 50.8 years; for further characteristics see Table 3). The vast majority (95.5%) came for a follow-up visit, and 99% currently received antipsoriatic treatment. 18.5% of the participants (n = 37) were treated exclusively with topical therapy, 58% (n = 116) with topical therapy in combination with another treatment modality, 10% (n = 20) with phototherapy, 37.5% (n = 75) with traditional systemic antipsoriatic medication and 43.5% (n = 87) with biologicals at the time of data collection. Therefore the mean PASI was relatively low (3.4, range: 0–26.7). The mean DLQI was 6.2 (range: 0–30), corresponding to moderate disease-related life quality impairment.

**Table 3:** Characteristics of the study cohort.

Category	N (%)^1^
**Gender**	
Female	85 (42.5)
Male	115 (57.5)
**Age (years)**	
Mean (SD)	50.8 (14.1)
Median (min-max; IQR)	51 (18–84; 17.8)
**Partnership**	
Living with a partner	121 (60.5)
Living without a partner	79 (39.5)
**Occupational status** ^2^	
Working full-time	102 (51)
Working part-time	30 (15)
Not working^3^	62 (31)
**Net monthly household income (€)** ^4^	
<1500	40 (20)
1500–3000	75 (37.5)
≥3000	39 (19.5)
**PASI**	
Mean (SD)	3.4 (4.1)
Median (min-max; IQR)	2 (0–26.7; 4.4)
**DLQI**	
Mean (SD)	6.2 (7.1)
Median (min-max; IQR)	4 (0–30; 9)
**Disease duration**	
Mean (SD)	19.9 (13.1)
Median (min-max; IQR)	19.5 (1–60; 21)
**Psoriatic arthritis**	45 (22.5)

^1^ Mean (SD) and median (min-max; IQR) are indicated for age, PASI and DLQI. N (%) are displayed for all other variables.

^2^ Information on the working status was unavailable for 6 participants (3%).

^3^ The subgroup of non-working participants comprised 51 retirees and 11 younger individuals who were either homemakers or unemployed.

^4^ 46 participants (23%) did not want to disclose their net monthly household income.

DLQI: Dermatology Life Quality Index; IQR: interquartile range; max: maximum; min: minimum; N: number; PASI: Psoriasis Area and Severity Index; SD: standard deviation.

### Patients’ preferences averaged across the study sample

**Fig 1:**
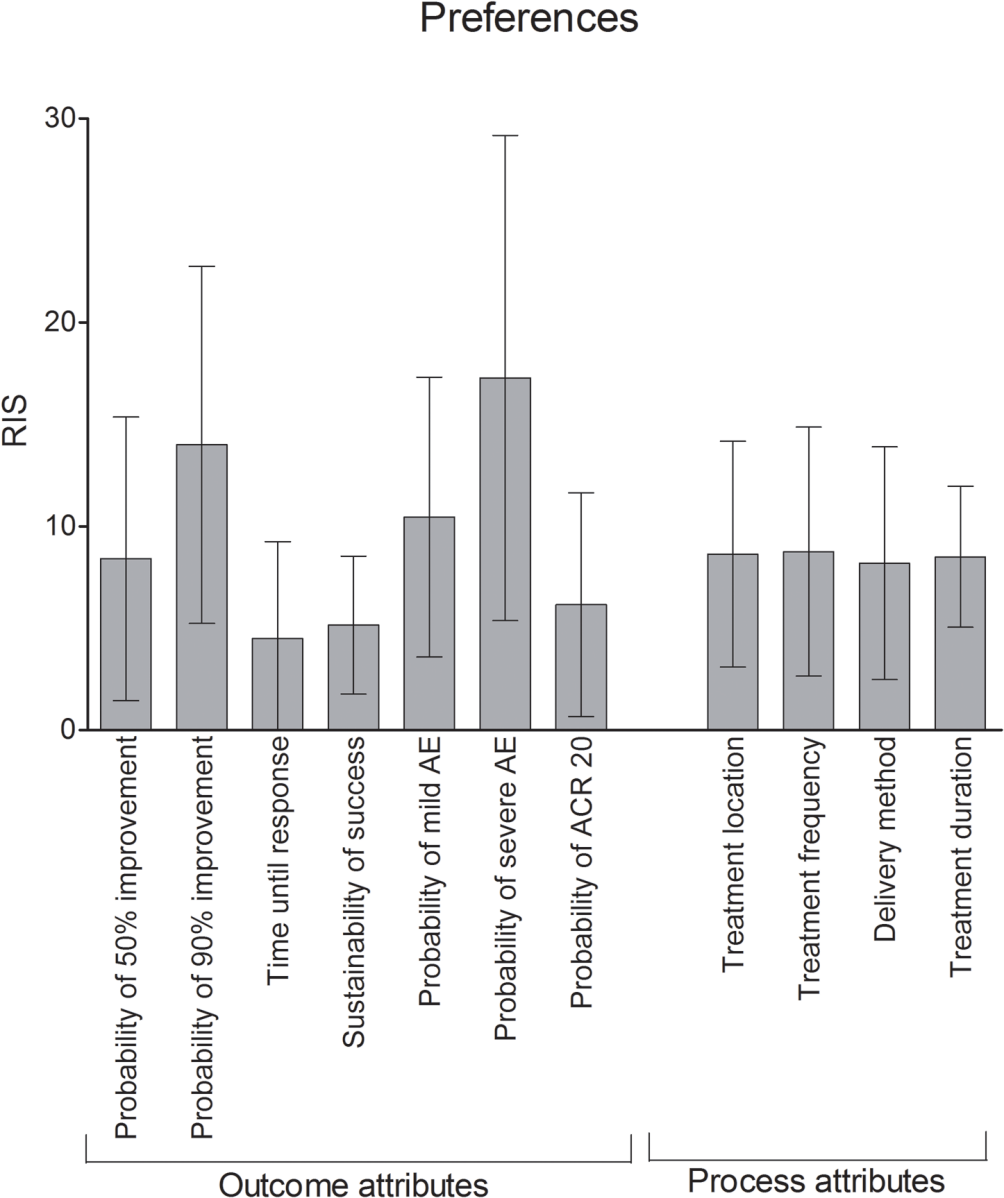
Relative Importance Scores (RIS) averaged across the study cohort. The probability of severe AE was evaluated as most important (RIS = 17.3), followed by the probability of 90% improvement (RIS = 14.0). Time until response (RIS = 4.5) and sustainability of the therapeutic success (RIS = 5.2) were least relevant. Bars: Means with standard deviations.

The attribute regarded as most important in the whole study collective was probability of severe AE (RIS = 17.3), followed by probability of 90% improvement (RIS = 14.0) and probability of mild AE (RIS = 10.5). Time until response (RIS = 4.5), sustainability of success (RIS = 5.2) and probability of ACR 20 response (RIS = 6.2) were rated less important. All process attributes reached RIS between 8.2 and 8.8 (Fig 1).

### Impact of sociodemographic and socioeconomic characteristics, PASI and DLQI on preferences

**Fig 2:**
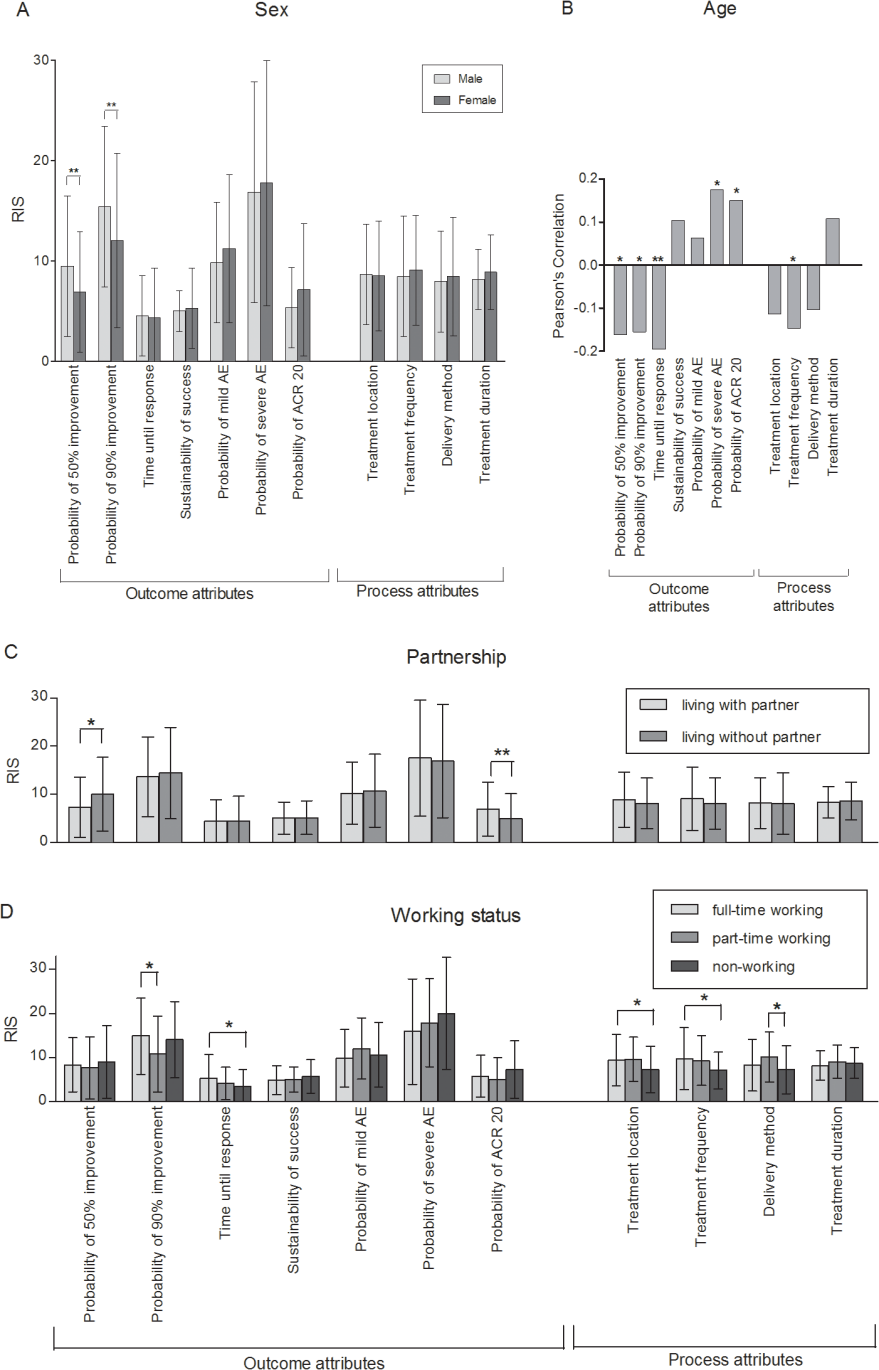
Impact of gender, age, partnership and working status on patients’ preferences. (A) Men attached higher value to the probability of 50% and 90% improvement than women. (B) Probability of 50% and 90% improvement, time until response and treatment frequency became less important with increasing age whereas probability of severe AE and probability of ACR 20 response gained relevance. (C) Participants without a partner placed greater importance on the probability of 50% improvement while respondents with a partner valued the probability of ACR 20 response higher. (D) Compared to non-working participants, full-time working participants set higher priority to time until response, treatment location, and treatment frequency. The probability of 90% improvement was more important for full-time working than for part-time working participants. Part-time working participants considered the delivery method more important than non-working participants. Differences in RIS were tested for significance with ANOVA (A, C), 2-tailed t-test (B) or Bonferroni post-hoc tests (D). Bars: Means with standard deviations (A, C, D) or Pearson’s Correlations (B). RIS: Relative Importance Scores. * p≤0.05, ** p≤0.01.

Subgroup analyses according to gender revealed that men were more concerned about the probability of 50% and 90% improvement than women (50% improvement: RIS = 9.5 vs. 6.9, p = 0.008; 90% improvement: RIS = 15.4 vs. 12.1, p = 0.002, Fig 2A). According to regression analyses these results were independent of age, partnership, working status, PASI and DLQI (50% improvement: β = -0.191, p = 0.011; 90% improvement: β = -0.197, p = 0.009, Table 4). Furthermore, models adjusted for these factors showed that women attached greater value to treatment frequency than men (β = 0.161, p = 0.035, Table 5).

**Table 4:** Multivariate linear regression models for outcome attributes.

Outcome attributes														
Characteristic	Probability of 50% improvement		Probability of 90% improvement		Time until response		Sustainability of success		Probability of mild AE		Probability of severe AE		Probability of ACR 20 response	
	β	p	β	p	β	p	β	p	β	p	β	p	β	p
**Female** ^1^	**-0.191**	**.011**	**-0.197**	**.009**	0.094	.221	-0.018	.823	0.054	.490	0.011	.883	0.135	.074
**Age**	**-0.219**	**.017**	**-0.264**	**.004**	-0.146	.123	0.088	.360	0.113	.236	0.166	.082	0.108	.245
**No partner** ^2^	**0.146**	**.044**	0.005	.948	-0.063	.402	0.023	.762	-0.014	.848	0.027	.719	**-0.196**	**.008**
**Part-time working** ^3^	0.003	.967	-0.129	.094	-0.065	.413	0.028	.723	0.108	.177	0.082	.304	-0.144	.064
**Not working** ^3^	0.12	.189	0.169	.067	-0.11	.246	0.082	.398	-0.032	.740	0.031	.749	-0.001	.994
**PASI**	-0.023	.77	-0.032	.684	-0.036	.658	0.012	.882	0.107	.188	0.032	.694	-0.006	.935
**DLQI**	**-0.168**	**.031**	0.024	.755	0.06	.456	0.08	.326	0.046	.567	-0.083	.304	0.08	.312

The RIS was defined as dependent variable; gender, age, partnership, working status, DLQI and PASI were used as independent variables. β represents the standardized regression coefficient. For metric variables (age, DLQI, PASI) a positive β-value indicates that the attribute gains importance with increase of the characteristic whereas a negative β-value indicates loss of importance of the attribute with increase of the characteristic. For all other variables a positive β indicates a higher importance of the attribute compared to the reference group. Statistically significant findings are highlighted in bold.

^1^ The reference group for “female” was male.

^2^ The reference group for “no partner” contained all participants living in a partnership.

^3^ The reference group for “part-time working” and “not working” comprised all participants working full-time.

DLQI: Dermatology Life Quality Index; PASI: Psoriasis Area and Severity Index; RIS: Relative Importance Score.

**Table 5:** Multivariate linear regression models for process attributes.

Process attributes								
Characteristic	Treatment location		Treatment frequency		Delivery method		Treatment duration	
	β	p	β	p	β	p	β	p
**Female** ^1^	0.01	.901	**0.161**	**.035**	0.02	.797	0.063	.409
**Age**	-0.023	.808	-0.086	.357	-0.029	.757	0.173	.066
**No partner** ^2^	-0.054	.473	-0.115	.123	-0.053	.482	-0.038	.613
**Part-time working** ^3^	0.015	.845	-0.051	.511	0.129	.106	0.065	.405
**Not working** ^3^	**-0.187**	**.051**	**-0.192**	**.042**	-0.093	.333	-0.029	.760
**PASI**	-0.08	.322	0.019	.811	-0.069	.393	0.107	.181
**DLQI**	-0.007	.933	-0.007	.931	0.044	.590	0.147	.066

For explanations and abbreviations, see Table 4.

^1^ The reference group for “female” was male.

^2^ The reference group for “no partner” contained all participants living in a partnership.

^3^ The reference group for “part-time working” and “not working” comprised all participants working full-time.

DLQI: Dermatology Life Quality Index; PASI: Psoriasis Area and Severity Index; RIS: Relative Importance Score.

Older participants judged the probability of 50% and 90% improvement less important than younger ones (50% improvement: PC = -0.161, p = 0.022; 90% improvement: PC = -0.155, p = 0.028, Fig 2B), findings substantiated in adjusted regression models (50% improvement: β = -0.219, p = 0.017; 90% improvement: β = -0.264, p = 0.004, Table 4). However, older participants worried more about severe AE (PC = 0.175, p = 0.013; β = 0.166, p = 0.082). According to bivariate analyses, ACR 20 response appeared more relevant (PC = 0.150, p = 0.035) but time until response (PC = -0.195, p = 0.006) and treatment frequency (PC = -0.146, p = 0.039) less relevant with increasing age (Fig 2B).

Participants living without a partner were more interested in 50% improvement than those with a partner (RIS = 10.1 vs. 7.3, p = 0.015, Fig 2C; β = 0.146, p = 0.044, Table 4). However, they were less concerned about ACR 20 response (RIS = 5.0 vs. 6.9; p = 0.004, β = -0.196, p = 0.008).

Monthly household income had no statistically significant impact on preferences, but preferences were influenced by working status (Fig 2D, Table 5). Probability of 90% improvement was more important for participants working full-time compared to those working part-time (RIS = 14.8 vs. 10.7, p = 0.033). Furthermore, participants with a full-time job were more interested in time until response (RIS = 3.4 vs. 5.2, p = 0.042), treatment location (RIS = 7.2 vs. 9.4, p = 0.026; β = -0.187, p = 0.051) and treatment frequency (RIS = 7.0 vs. 9.7, p = 0.018; β = 0.192, p = 0.042) than non-working participants. Participants working part-time were more concerned about the delivery method than those without work (RIS = 7.2 vs. 10.0, p = 0.016).

**Fig 3:**
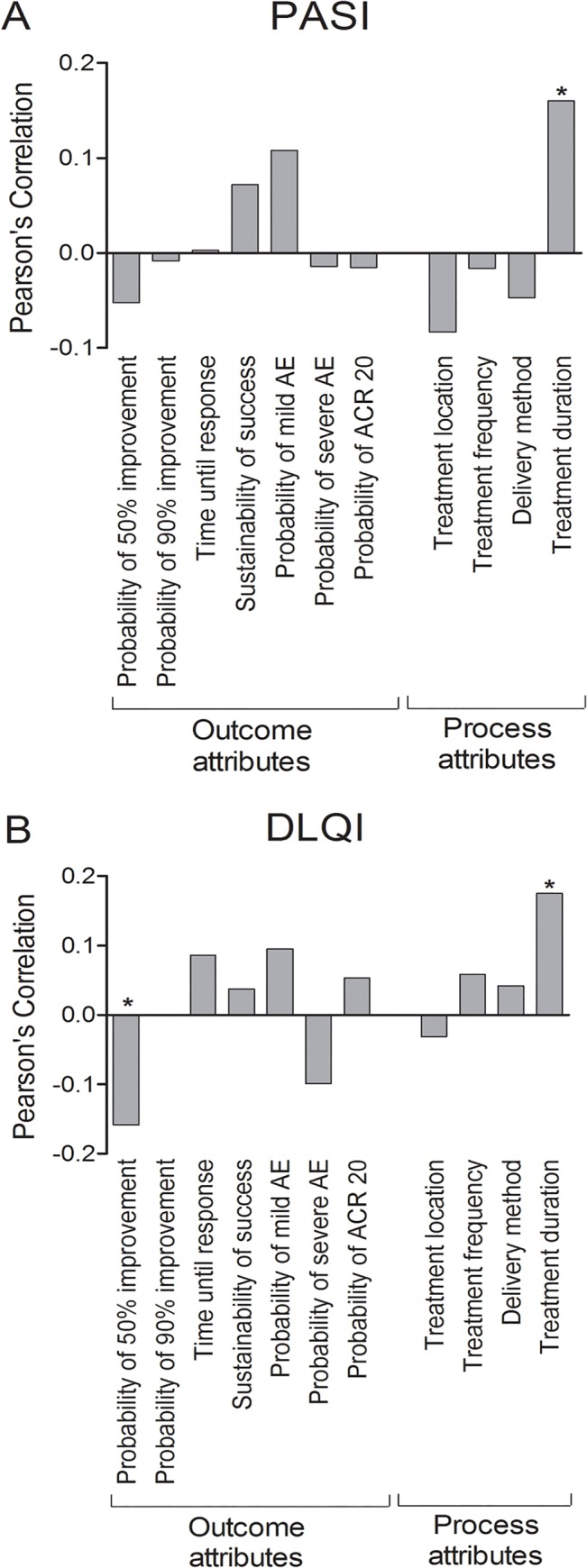
Impact of PASI and DLQI on Relative Importance Scores (RIS). With increasing PASI (A) and increasing DLQI (B), participants set greater value on treatment duration. The higher the DLQI score, the less importance was attached to probability of 50% improvement. Differences in RIS were tested for significance with 2-tailed t-tests. Bars: Pearson’s Correlations. * p≤0.05.

Treatment duration became more important with rising PASI (PC = 0.160, p = 0.024, Fig 3A) and rising DLQI (PC = 0.175, p = 0.013, Fig 3B; β = 0.147, p = 0.066, Table 5). Unexpectedly, the RIS for probability of 50% improvement was negatively correlated with DLQI (PC = -0.158, p = 0.026, Fig 3B), indicating that participants with higher disease-related quality of life impartment were less interested in reduction of their psoriasis by half. This finding was confirmed in multivariate regression models (β = -0.168, p = 0.031, Table 4).

## Discussion

In our study we applied a method closely resembling clinical decision-making for the identification of patients’ preferences for biologicals. Attributes and attribute levels reflected characteristics of biologicals approved for psoriasis in Germany as accurately as possible in order to create choice scenarios close to reality. We show that participants attach the greatest importance to avoidance of severe AE, in line with CA from Seston et al., according to which psoriasis patients were willing to trade time until response for a reduced risk of severe AE, particularly skin cancer and liver damage [16].

We previously performed CA to assess patients’ preferences for all treatment options currently available for psoriasis [18]. In these experiments patients prioritized process attributes over outcome. The attribute regarded as most important was treatment location, probably because participants were confronted with the option of a three-week hospitalization for treatment with dithranol, which they disliked [18]. Under the prerequisite of an outpatient setting and given the overall convenient treatment process associated with biologicals, participants care only moderately about the treatment process. Regarding the delivery method, our part-worth utilities indicated than an injection pen was most preferred, followed by subcutaneous syringes, a finding well in accordance with other studies (e.g., [27]).

The relevance attributed to AE can be largely influenced by the examples given, because patients tend to imagine these conditions and emphasize them during their decision-making, a phenomenon called the framing effect [28]. In our previous study comparing preferences for all treatment modalities, utilities for AE were assessed without giving specific examples, since none of them could have applied to all options at once, and RIS for AE-related attributes were low. Here we included examples of several important mild and severe AE of biologicals. As the amount of information had to be limited, it was impossible to mention each potential AE. In addition, our study does not allow conclusions on how the different kinds of AE can influence preferences. The perception of rare AE can vary immensely depending on how they are presented to the patients. When weighing AE against efficacy, it has to be taken into account that the mean PASI of our participants was relatively low. Thanks to their actually good disease control, participants might have focused more on AE than on clearing.

Regarding efficacy, we assessed utilities of 50% and 90% improvement instead of PASI 75 response rate, which is the most common primary endpoint of clinical trials. First, 50% and 90% improvement is easier to imagine for study participants than PASI 75 response. Second, 90% improvement rates of currently available biologicals, roughly reflecting PASI 90 responses, differ stronger than their PASI 75 response rates. Third, we wanted to examine whether 50% improvement is still a worthwhile treatment goal for patients. Not surprisingly, probability of 90% improvement was identified as the second most important attribute. Efficacy is a key predictor of satisfaction with antipsoriatic medications [12,29]. High prioritization of 90% improvement may indicate that in the era of biologicals patients’ expectations are rising towards (nearly) complete clearance. However, participants still valued 50% improvement.

When stratifying the RIS for gender, men put higher emphasis on treatment efficiency. This result is surprising, considering that women usually attach greater importance to appearance [30] and suffer more from their psoriasis than men, both mentally and physically [31].

Older study participants were willing to trade efficacy and rapidity of response for safety, corresponding to our previous observations [18]. Indeed, older patients have a higher risk of concomitant diseases and AE from medication [32,33]. Therefore, they might be unwilling to accept additional risks of systemic antipsoriatic treatments.

Participants living without a partner valued 50% improvement higher, possibly due to expectations from society and potential partners [18]. However, they were less interested in ACR 20 response. The prevalence of psoriatic arthritis rises with increasing age and duration of psoriasis. Participants without a partner were in average younger than those with a partner (mean age: 46.9 vs. 53.3 years) but the difference in utilities of ACR 20 response was still statistically significant in multivariate models controlling for age.

Participants working full-time put emphasis on a treatment well compatible with work, i.e., on high probability of 90% improvement, rapid onset of action, low treatment frequency, and a convenient treatment location. Psoriasis can substantially impair work productivity, [34,35] and working patients might feel pressure to reduce absenteeism. The subgroup of participants who were not working comprised homemakers, unemployed and retired participants. It is well conceivable that preferences of homemakers and retired persons differ from those of unemployed patients. Accordant analyses were impossible here due to the study design and the limited cohort size but will be interesting to perform in future larger-scale studies.

The study cohort was heterogeneous with regard to PASI and DLQI and the mean PASI was relatively low, but these parameters were indicative of disease control and not of global disease severity, since virtually all participants received treatment at the time of study participation. Participants with higher PASI and higher DLQI were particularly interested in a time-saving treatment.

A limitation of our study is that it only comprised patients with moderate-to-severe psoriasis treated at a German University Hospital. Clearly, our findings will have to be verified in larger cohorts and in a multi-centric setting. Moreover, it is likely that preferences can be influenced by further factors such as comorbidities and treatment experience. For example, patients with psoriatic arthritis are more interested in ACR 20 response than others (Schaarschmidt et al., manuscript in preparation). 46% of the participants had experience with biologicals, but all were candidates for these medications and may be confronted with treatment decisions involving biologicals.

A major limitation of conjoint analysis is that the discrete choice experiments are theoretical and actual patients may choose actual medications differently. Moreover, average preferences presented here do not allow direct conclusions for each individual. Clearly, treatment decisions for a particular patient are based on his or her individual preferences. However, when starting to discuss treatment options with an individual, it is extremely helpful for physicians to know what most patients are interested in and concerned about and how preferences may be systematically influenced by sociodemographic and disease-related characteristics. Based on this knowledge, physicians should work out the preferences, needs and concerns of each individual patient and integrate them into therapeutic decisions in order to optimize treatment satisfaction, adherence and outcome.

## References

[1] de Korte J, Sprangers MA, Mombers FM, Bos JD. Quality of life in patients with psoriasis: a systematic literature review. J Investig Dermatol Symp Proc. 2004; 9: 140–147. 15083781 10.1046/j.1087-0024.2003.09110.x

[2] Blome C, Simianer S, Purwins S, Laass A, Rustenbach SJ, Schaefer I, et al Time needed for treatment is the major predictor of quality of life in psoriasis. Dermatology. 2010; 221: 154–159. 10.1159/000313825 20558972

[3] Dubertret L, Mrowietz U, Ranki A, van de Kerkhof PC, Chimenti S, Lotti T, et al European patient perspectives on the impact of psoriasis: the EUROPSO patient membership survey. Br J Dermatol. 2006; 155: 729–736. 16965422 10.1111/j.1365-2133.2006.07405.x

[4] Menter A, Gottlieb A, Feldman SR, Van Voorhees AS, Leonardi CL, Gordon KB, et al Guidelines of care for the management of psoriasis and psoriatic arthritis: Section 1. Overview of psoriasis and guidelines of care for the treatment of psoriasis with biologics. J Am Acad Dermatol. 2008; 58: 826–850. 10.1016/j.jaad.2008.02.039 18423260

[5] Leonardi CL, Kimball AB, Papp KA, Yeilding N, Guzzo C, Wang Y, et al Efficacy and safety of ustekinumab, a human interleukin-12/23 monoclonal antibody, in patients with psoriasis: 76-week results from a randomised, double-blind, placebo-controlled trial (PHOENIX 1). Lancet. 2008; 371: 1665–1674. 10.1016/S0140-6736(08)60725-4 18486739

[6] Reich K, Burden AD, Eaton JN, Hawkins NS. Efficacy of biologics in the treatment of moderate to severe psoriasis: a network meta-analysis of randomized controlled trials. Br J Dermatol. 2012; 166: 179–188. 10.1111/j.1365-2133.2011.10583.x 21910698

[7] Gniadecki R, Bang B, Bryld LE, Iversen L, Lasthein S, Skov L. Comparison of long-term drug survival and safety of biologic agents in patients with psoriasis vulgaris. Br J Dermatol. 2015; 172: 244–252. 10.1111/bjd.13343 25132294

[8] Nast A, Sporbeck B, Rosumeck S, Pathirana D, Jacobs A, Werner RN, et al Which antipsoriatic drug has the fastest onset of action? Systematic review on the rapidity of the onset of action. J Invest Dermatol. 2013; 133: 1963–1970. 10.1038/jid.2013.78 23426133

[9] Ash Z, Gaujoux-Viala C, Gossec L, Hensor EM, FitzGerald O, Winthrop K, et al A systematic literature review of drug therapies for the treatment of psoriatic arthritis: current evidence and meta-analysis informing the EULAR recommendations for the management of psoriatic arthritis. Ann Rheum Dis. 2012; 71: 319–326. 10.1136/ard.2011.150995 21803753

[10] McInnes IB, Kavanaugh A, Gottlieb AB, Puig L, Rahman P, Ritchlin C, et al Efficacy and safety of ustekinumab in patients with active psoriatic arthritis: 1 year results of the phase 3, multicentre, double-blind, placebo-controlled PSUMMIT 1 trial. Lancet. 2013; 382: 780–789. 10.1016/S0140-6736(13)60594-2 23769296

[11] Augustin M, Holland B, Dartsch D, Langenbruch A, Radtke MA. Adherence in the treatment of psoriasis: a systematic review. Dermatology. 2011; 222: 363–374. 10.1159/000329026 21757881

[12] van Cranenburgh OD, de Korte J, Sprangers MA, de Rie MA, Smets EM. Satisfaction with treatment among patients with psoriasis: a web-based survey study. Br J Dermatol. 2013; 169: 398–405. 10.1111/bjd.12372 23565643

[13] Opmeer BC, Heydendael VM, deBorgie CA, Spuls PI, Bossuyt PM, Bos JD, et al Patients with moderate-to-severe plaque psoriasis preferred oral therapies to phototherapies: a preference assessment based on clinical scenarios with trade-off questions. J Clin Epidemiol. 2007; 60: 696–703. 17573985 10.1016/j.jclinepi.2006.10.011

[14] Kjaer T, Bech M, Gyrd-Hansen D, Hart-Hansen K. Ordering effect and price sensitivity in discrete choice experiments: need we worry? Health Econ. 2006; 15: 1217–1228. 16786550 10.1002/hec.1117

[15] Ashcroft DM, Seston E, Griffiths CE. Trade-offs between the benefits and risks of drug treatment for psoriasis: a discrete choice experiment with U.K. dermatologists. Br J Dermatol. 2006; 155: 1236–1241. 17107395 10.1111/j.1365-2133.2006.07535.x

[16] Seston EM, Ashcroft DM, Griffiths CE. Balancing the benefits and risks of drug treatment: a stated-preference, discrete choice experiment with patients with psoriasis. Arch Dermatol. 2007; 143: 1175–1179. 17875880 10.1001/archderm.143.9.1175

[17] Umar N, Yamamoto S, Loerbroks A, Terris D. Elicitation and use of patients' preferences in the treatment of psoriasis: a systematic review. Acta Derm Venereol. 2012; 92: 341–346. 10.2340/00015555-1304 22278662

[18] Schaarschmidt ML, Schmieder A, Umar N, Terris D, Goebeler M, Goerdt S, et al Patient preferences for psoriasis treatments: process characteristics can outweigh outcome attributes. Arch Dermatol. 2011; 147: 1285–1294. 10.1001/archdermatol.2011.309 22106115

[19] Schaarschmidt ML, Umar N, Schmieder A, Terris D, Goebeler M, Goerdt S, et al Patient preferences for psoriasis treatments: impact of treatment experience. J Eur Acad Dermatol Venereol. 2013; 27: 187–198. 10.1111/j.1468-3083.2011.04440.x 22225546

[20] Schmieder A, Schaarschmidt ML, Umar N, Terris DD, Goebeler M, Goerdt S, et al Comorbidities significantly impact patients' preferences for psoriasis treatments. J Am Acad Dermatol. 2012; 67: 363–372. 10.1016/j.jaad.2011.08.023 22015150

[21] Umar N, Schaarschmidt M, Schmieder A, Peitsch WK, Schöllgen I, Terris DD. Matching physicians' treatment recommendations to patients' treatment preferences is associated with improvement in treatment satisfaction. J Eur Acad Dermatol Venereol. 2013; 27: 763–770. 10.1111/j.1468-3083.2012.04569.x 22631875

[22] Taylor W, Gladman D, Helliwell P, Marchesoni A, Mease P, Mielants H, et al Classification criteria for psoriatic arthritis: development of new criteria from a large international study. Arthritis Rheum. 2006; 54: 2665–2673. 16871531 10.1002/art.21972

[23] Reich K, Sinclair R, Roberts G, Griffiths CE, Tabberer M, Barker J. Comparative effects of biological therapies on the severity of skin symptoms and health-related quality of life in patients with plaque-type psoriasis: a meta-analysis. Curr Med Res Opin. 2008; 24: 1237–1254. 10.1185/030079908X291985 18355421

[24] Schmitt J, Zhang Z, Wozel G, Meurer M, Kirch W. Efficacy and tolerability of biologic and nonbiologic systemic treatments for moderate-to-severe psoriasis: meta-analysis of randomized controlled trials. Br J Dermatol. 2008; 159: 513–526. 10.1111/j.1365-2133.2008.08732.x 18627372

[25] van Lumig PP, Driessen RJ, Berends MA, Boezeman JB, van de Kerkhof PC, de Jong EM. Safety of treatment with biologics for psoriasis in daily practice: 5-year data. J Eur Acad Dermatol Venereol. 2012; 26: 283–291. 10.1111/j.1468-3083.2011.04044.x 21435026

[26] Nast A, Boehncke WH, Mrowietz U, Ockenfels HM, Philipp S, Reich K, et al S3—Guidelines on the treatment of psoriasis vulgaris (English version). Update. J Dtsch Dermatol Ges. 2012; 10 Suppl 2: S1–95. 10.1111/j.1610-0387.2012.07919.x 22386073

[27] Paul C, Stalder JF, Thaci D, Vincendon P, Brault Y, Kielar D, et al Patient satisfaction with injection devices: a randomized controlled study comparing two different etanercept delivery systems in moderate to severe psoriasis. J Eur Acad Dermatol Venereol. 2012; 26: 448–455. 10.1111/j.1468-3083.2011.04093.x 21557778

[28] Gong J, Zhang Y, Yang Z, Huang Y, Feng J, Zhang W. The framing effect in medical decision-making: a review of the literature. Psychol Health Med. 2013; 18: 645–653. 10.1080/13548506.2013.766352 23387993

[29] Finlay AY, Ortonne JP. Patient satisfaction with psoriasis therapies: an update and introduction to biologic therapy. J Cutan Med Surg. 2004; 8: 310–320. 15868312 10.1007/s10227-005-0030-6

[30] Harris DL, Carr AT. Prevalence of concern about physical appearance in the general population. Br J Plast Surg. 2001; 54: 223–226. 11254414 10.1054/bjps.2001.3550

[31] Grozdev I, Kast D, Cao L, Carlson D, Pujari P, Schmotzer B, et al Physical and mental impact of psoriasis severity as measured by the compact Short Form-12 Health Survey (SF-12) quality of life tool. J Invest Dermatol. 2012; 132: 1111–1116. 10.1038/jid.2011.427 22205305 PMC3366426

[32] Hovstadius B, Hovstadius K, Astrand B, Petersson G. Increasing polypharmacy—an individual-based study of the Swedish population 2005–2008. BMC Clin Pharmacol. 2010; 10: 16 10.1186/1472-6904-10-16 21122160 PMC3014875

[33] Boyd CM, Ritchie CS, Tipton EF, Studenski SA, Wieland D. From Bedside to Bench: summary from the American Geriatrics Society/National Institute on Aging Research Conference on Comorbidity and Multiple Morbidity in Older Adults. Aging Clin Exp Research. 2008; 20: 181–188. 18594183 10.1007/bf03324775 PMC3104206

[34] Armstrong AW, Robertson AD, Wu J, Schupp C, Lebwohl MG. Undertreatment, treatment trends, and treatment dissatisfaction among patients with psoriasis and psoriatic arthritis in the United States: findings from the National Psoriasis Foundation surveys, 2003–2011. JAMA Dermatol. 2013; 149: 1180–1185. 10.1001/jamadermatol.2013.5264 23945732

[35] Kimball AB, Yu AP, Signorovitch J, Xie J, Tsaneva M, Gupta SR, et al The effects of adalimumab treatment and psoriasis severity on self-reported work productivity and activity impairment for patients with moderate to severe psoriasis. J Am Acad Dermatol. 2012; 66: e67–76. 10.1016/j.jaad.2010.10.020 21616560

